# How female × male and male × male interactions influence competitive fertilization in *Drosophila melanogaster*

**DOI:** 10.1002/evl3.193

**Published:** 2020-09-04

**Authors:** Stefan Lüpold, Jonathan Bradley Reil, Mollie K. Manier, Valérian Zeender, John M. Belote, Scott Pitnick

**Affiliations:** ^1^ Department of Evolutionary Biology and Environmental Studies University of Zurich Zurich CH‐8057 Switzerland; ^2^ Department of Biology Syracuse University Syracuse New York 13244; ^3^ Department of Entomology Cornell University Ithaca New York 14853; ^4^ Department of Plant and Environmental Protection Sciences University of Hawaii at Mānoa Honolulu Hawaii 96822; ^5^ Department of Biological Sciences George Washington University Washington DC 20052

**Keywords:** Cryptic female choice, ejaculate‐female interactions, female reproductive tract, genetic compatibility, postcopulatory sexual selection, sperm competition, trait diversification

## Abstract

How males and females contribute to joint reproductive success has been a long‐standing question in sexual selection. Under postcopulatory sexual selection, paternity success is predicted to derive from complex interactions among females engaging in cryptic female choice and males engaging in sperm competition. Such interactions have been identified as potential sources of genetic variation in sexually selected traits but are also expected to inhibit trait diversification. To date, studies of interactions between females and competing males have focused almost exclusively on genotypes and not phenotypic variation in sexually selected traits. Here, we characterize within‐ and between‐sex interactions in *Drosophila melanogaster* using isogenic lines with heritable variation in both male and female traits known to influence competitive fertilization. We confirmed, and expanded on, previously reported genotypic interactions within and between the sexes, and showed that several reproductive events, including sperm transfer, female sperm ejection, and sperm storage, were explained by two‐ and three‐way interactions among sex‐specific phenotypes. We also documented complex interactions between the lengths of competing males’ sperm and the female seminal receptacle, which are known to have experienced rapid female‐male co‐diversification. Our results highlight the nonindependence of sperm competition and cryptic female choice and demonstrate that complex interactions between the sexes do not limit the ability of multivariate systems to respond to directional sexual selection.

**Impact Summary**

For species with internal fertilization and female promiscuity, postcopulatory sexual selection (PSS) is believed to depend, in part, on complex interactions between rival males and between the sexes. Although little investigated, clarifying such interactions is critical as they may limit the efficacy of PSS in the diversification of reproductive traits (e.g., ejaculate biochemistry and sperm, genitalia and female reproductive tract morphology). Here, we resolve how sex‐specific traits and their interactions contribute to key reproductive events and outcomes related to competitive fertilization success, including traits known to have experienced rapid diversification. Our results provide novel insights into the operation and complexity of PSS and demonstrate that the processes of sperm competition and cryptic female choice are not independent selective forces. Simultaneously, the complex interactions between the sexes do not necessarily limit rapid trait diversification in multivariate systems.

Because females of most species mate with multiple males within reproductive cycles (Arnqvist and Rowe 2005; Taylor et al. 2014), sexual selection, encompassing both male‐male competition and female choice, can continue after mating in the form of sperm competition and cryptic female choice, respectively (Parker 1970a; Eberhard 1996). As for premating sexual selection, a longstanding goal of studies of postcopulatory sexual selection (PSS) has been to characterize genetic variation in male traits and female preferences that are believed to be the targets of sexual selection, and to identify how such variation relates to differential reproductive success. Explicit demonstration of the means by which phenotypes affect fitness is a prerequisite for resolving the selective processes (Endler 1986; Sober 1993). However, compared to premating sexual selection (Andersson 1994; Jennions et al. 2001; Kokko and Jennions 2003), our causal, mechanistic understanding of how variation in postcopulatory sexual traits translates into fitness, and so how PSS contributes to trait diversification, is relatively scant (Howard et al. 2009; Lüpold and Pitnick 2018).

Our understanding of PSS is limited partly due to challenges of observing its processes within the female reproductive tract (FRT) and of discriminating between competing sperm (Manier et al. 2010, 2013a,b). However, two further, interrelated aspects of PSS cloud our understanding of its role in both maintaining genetic variation and driving evolutionary diversification. First, sex‐specific mediators of competitive fertilization success tend to be multivariate, potentially including a multitude of genitalic, seminal fluid, sperm, and FRT morphological, physiological, neurological, and/or biochemical traits, any of which may influence sperm transfer, storage, maturation, motility, longevity, and contribution to fertilization (Snook 2005; Poiani 2006; Pitnick et al. 2009a,b; Carmel et al. 2016). Second, because sperm competition takes place within the FRT, the competitiveness of ejaculates is likely to depend in large measure on their interactions with the female (Ravi Ram and Wolfner 2007; Pitnick et al. 2009b, 2020; Sirot and Wolfner 2015). Any variation in the FRT environment may change the conditions under which sperm compete, and therefore, shift the relative competitive advantage between males (Eberhard 1996; Firman et al. 2017; Lüpold and Pitnick 2018). That such female × male interactions can influence patterns of sperm precedence has been demonstrated in diverse species with both internal (Lewis and Austad 1990; Wilson et al. 1997; Clark et al. 1999; Miller and Pitnick 2002; Nilsson et al. 2003; Birkhead et al. 2004; Chow et al. 2010; Delbare et al. 2017) and external fertilization (Turner and Montgomerie 2002; Evans and Marshall 2005; Rosengrave et al. 2008; Simmons et al. 2009; Alonzo et al. 2016). Further evidence for such interactions comes from studies of conspecific sperm precedence (Howard et al. 2009; Manier et al. 2013a,b,c). In fact, mounting evidence suggests that competitive fertilization events may rarely be independent of female effects (Eberhard 1996; Lüpold et al. 2016).

Considering these interactions among multivariate traits and between sexes, PSS is expected to favor the maintenance of genetic variation in reproductive characters and to inhibit strong directional selection on specific traits for three reasons. First, having numerous traits contributing to a fitness outcome can dilute the strength of selection on any single trait. Second, directional sexual selection on males should be limited if their mating or competitive fertilization success is influenced by their compatibility with females rather than their intrinsic quality (Birkhead 1998; Pitnick and Brown 2000; Tregenza and Wedell 2000; Neff and Pitcher 2005; Oh and Badyaev 2006). Third, many interacting traits provide the requisite conditions for nontransitive competitive outcomes in the manner of a rock‐paper‐scissors game (Maynard Smith 1982), which further limits the strength of directional selection (Clark 2002). Nontransitivity in competitive fertilization success has been experimentally demonstrated for *Drosophila melanogaster* by using fixed‐chromosome lines (Clark et al. 1999, 2000; Zhang et al. 2013; Reinhart et al. 2015), and for domestic fowl (*Gallus gallus domesticus*) by using artificial insemination (Birkhead et al. 2004).

Genotypic interactions between the sexes could be pervasive or even ubiquitous. If true, then we predict that even where competitive fertilization success is determined purely by raffle‐based sperm competition, it would function as a loaded raffle (Parker 1990) due to differential compatibility between each male's sperm and the FRT (e.g., Pitnick et al. 2020). This assumption is the basis for the contention that, relative to premating sexual selection, PSS intrasexual competition and intersexual choice are a false dichotomy and fall more on a continuum (e.g., Eberhard 1996; Arnqvist 2014; Lüpold et al. 2016; Lüpold and Pitnick 2018). The position of the two selective processes along this continuum is likely to be determined by the relative contributions of sex‐specific effects to three‐way interactions between physically or biochemically interacting traits of females and competing males. However, without a detailed understanding of the underlying processes it is near impossible to determine whether variation in reproductive outcomes between females and competing males is primarily attributable to, for example, genotype‐ or condition‐dependent postcopulatory female biases toward certain sperm phenotypes or differential performance of competing sperm within these different selective environments. Hence, unlike male‐male contest competition, for example, where males may compete over access to mating opportunities even before females arrive, male‐male competition at the postcopulatory stage would rarely escape direct female involvement, particularly in internal fertilizers. Due to the predicted interactions between females and males, we also see great potential for the same, or tightly linked, ejaculate traits to be favored by both inter‐ and intrasexual processes of selection as sperm would be most competitive in a selective environment that is more favorable to them.

In an alternative scenario, ejaculate‐female interactions could be more limited in scope, for example, by being restricted to processes of cryptic female choice. If so, we would predict such limitation to contribute to the discreteness of processes underlying sperm competition and cryptic female choice, respectively, and that traits of sperm competition should have a greater potential to respond to directional sexual selection than those of cryptic female choice. If these selective processes are largely independent rather than intertwined as described above, there should, at least in principle, also be greater scope for intra‐ and intersexual selection to target separate ejaculate traits, even though ejaculate traits may rarely evolve independently (Gómez Montoto et al. 2011; Fitzpatrick et al. 2012; Lüpold 2013; Lymbery et al. 2018; Liao et al. 2019).

To date, it has been impossible to empirically disentangle predictions about the relative importance of sperm competition and cryptic female choice, or about the extent to which they are discrete selective processes. The main impediments in such exploration are that all studies demonstrating female × male interactions in the pattern of sperm precedence or competitive fertilization success have done so without investigating any specific traits (i.e., by using genetically discrete lines or geographic populations), or else have examined only a single pair of interacting, sex‐specific traits, such as sperm length and female seminal receptacle (SR) length (Miller and Pitnick 2002) or genetic variation in male sex peptide and its female receptor (Zhang et al. 2013). Knowledge of the sex‐specific traits contributing to PSS processes and of the degree to which interactions between competing males and the females, respectively, mediate those processes is too limited for any system to make clear predictions about their influence on diversification and the maintenance of variation.

To advance our understanding of PSS, we set the goals of (1) identifying many of the putative sex‐specific targets of PSS (e.g., copulation duration; the number, length, and in vivo swimming velocity of sperm; female remating interval; fecundity; sperm‐storage organ morphometry; and sperm storage, ejection, and use), (2) quantifying their genetic variation, and (3) determining their contribution to variation in competitive fertilization success within a multivariate framework. To accomplish these goals, we embarked on a three‐stage research program using isogenic populations of *D. melanogaster* with sperm that express either green (GFP) or red (RFP) fluorescent protein in their sperm heads (Manier et al. 2010; Lüpold et al. 2012, 2013; Ala‐Honkola et al. 2013). The fluorescent tags allow direct visualization of living sperm within the FRT while discriminating between sperm from competing males within twice‐mated females, as well as tracking the spatiotemporal fate of both males’ sperm throughout remating and progeny production by females. In the first stage of this program, we held the female genetic background (i.e., isoline) constant and competed males from different isolines to resolve male‐mediated contributions to competitive fertilization success (Lüpold et al. 2012). We demonstrated that longer and slower sperm are better at displacing sperm of a previous male from the female SR (i.e., primary sperm‐storage organ), or resisting such displacement by incoming sperm of a subsequent mate (Lüpold et al. 2012). In the second stage, we resolved female‐mediated contributions by holding the genetic background of all males constant while competing their ejaculates within females from different isolines (Lüpold et al. 2013). This study revealed how females can strongly bias sperm storage between males by varying the time between remating and ejecting a mass containing excess second‐male and displaced first‐male sperm from their bursa copulatrix before initiating oviposition (Lüpold et al. 2013). Because in *D. melanogaster* paternity is shared among males in proportion to their sperm representation within the SR (Civetta 1999; Manier et al. 2010, 2013c; Lüpold et al. 2012, 2013), female sperm ejection is a key element of cryptic female choice in this species (Snook and Hosken 2004; Lüpold et al. 2013). Here, in the third and final installment, we report on experiments in which the genetic backgrounds of both competing males and females were systematically varied to identify genotypic effects and interactions while resolving the contribution of multivariate traits to female × male, male × male, and female × male × male interactions to variation in competitive fertilization success. Specifically, after competing different male genotypes in different female genetic backgrounds, we examined how the interactions between male (e.g., sperm length and number) and female attributes (e.g., remating interval or SR length) influence reproductive events known to affect competitive fertilization (e.g., timing of female postmating sperm ejection or sperm storage).

## Materials and Methods

### EXPERIMENTAL MATERIAL

We performed all experiments with LH_m_ populations of *D. melanogaster* that express a protamine labeled with either GFP or RFP in sperm heads (Manier et al. 2010), which permit discriminating sperm from different males and quantifying sperm within the FRT. Using random individuals from large population cages (all backcrossed to the LH_m_ wild type for six generations; Manier et al. 2010), we generated isogenic lines (“isolines”; Parsons and Hosgood 1968; David et al. 2005) by 15 generations of full‐sibling inbreeding, thus yielding theoretical homozygosity levels of 96% (Falconer 1989). To avoid inbreeding effects, we crossed independent pairs of isogenic lines (i.e., males of one isoline with virgin females of another) to create repeatable heterozygous F_1_ genotypes for the experiments described here. Male and female reproductive traits were previously characterized for these crosses and shown to be heritable, and replicated mating trials within given genotype combinations generate repeatable results (Lüpold et al. 2012, 2013, 2016). Based on these assays, we selected parental isolines that captured most of the variance in both male and female reproductive traits among genotypes when creating heterozygotes. In total, our experimental population consisted of six female RFP genotypes, an independent set of six first‐male RFP genotypes, and three second‐male GFP genotypes. We reared all flies at low densities in replicate vials with standard cornmeal‐molasses‐agar medium supplemented with yeast, collected them as virgins upon eclosion, and aged them for 3 (males) or 4 days (females) before their first mating. All males were mated once to a nonexperimental female on the day before their first experimental mating to avoid sexual naiveté (Bjork et al. 2007).

### SPERM COMPETITION EXPERIMENT

We have repeatedly shown that paternity (i.e., P_2_) in *D. melanogaster* (including in these isogenic lines) is directly proportional to the respective numbers of sperm from two competing males remaining in storage (S_2_), particularly within the SR, after females have ejected any excess second‐male and displaced first‐male sperm, thus following a fair raffle among stored sperm (Manier et al. 2010, 2013c; Lüpold et al. 2012, 2013). Therefore, we focused our efforts on investigating how female × male, male × male, and female × male × male interactions influence the process of sperm displacement until it is interrupted by female sperm ejection (Manier et al. 2010, 2013b; Lüpold et al. 2013) and used S_2_ as a proxy of P_2_. Throughout the text, we refer to S_2_ within the SR as the “fertilization set” (Parker 1990).

Within each of eight replicates, examined in four temporal blocks of two full replicate sets (staggered by 2 days), we mated virgin females each to an RFP male and, 2 days later, to a GFP male in all 108 possible combinations between genotypes (total *N* = 864 trios tested). Females not remating within 4 hours were given additional 4‐hour remating opportunities on days 3–5 after the first mating. Immediately after the end of the second mating, we removed the male from the mating vial, isolated the female in a glass three‐well spot plate beneath a glass coverslip, and checked for sperm ejection every 10 min for up to 5 hours using a stereomicroscope. We recorded the time to sperm ejection, immediately removed the female from the well and froze it for later quantification of stored sperm, and transferred the ejected mass to phosphate‐buffered saline (PBS) on a microscope slide and sealed the coverslip with rubber cement.

For all dissected females, we counted the sperm of both competitors across the different organs of the FRT (bursa copulatrix, SR, and paired spermathecae with ducts) and determined the total number of sperm for each male in all female sperm‐storage organs combined, and the proportion of total sperm derived from the second male (S_2_) across all storage organs and within the SR, respectively. Combining these counts with those of the ejected masses further permitted calculating the number of first‐male resident sperm at the time of remating, the number of second‐male sperm transferred, and both the absolute and relative number of each male's sperm stored and ejected, respectively.

Finally, for each of the nine male genotypes, we dissected six males after measuring their thorax length, retrieved sperm from their seminal vesicles using a fine probe, fixed the sample on a microscope slide with a mixture of methanol and acetic acid (3:1 [v/v]), rinsed it with PBS, and mounted it under a coverslip in glycerol and PBS (80:20 [v/v]). We measured the length of five sperm per male using the segmented line tool in ImageJ version 1.47 at 200× magnification under the dark‐field optics of an Olympus BX‐60 microscope. For each of the six female genotypes, we measured the thorax length of each of eight females, and dissected their reproductive tract into PBS on a microscope slide and covered it with a glass coverslip with clay at the corners, allowing the SR to be flattened to two dimensions without stretching. We then measured SR length using ImageJ at 200× magnification under an Olympus BX‐60 microscope with Nomarski DIC optics. Both sperm and SR length are significantly heritable (Miller and Pitnick 2002; Lüpold et al. 2012, 2013, 2016).

### STATISTICAL ANALYSES

We performed all analyses using the statistical software package R version 3.4.3 (R Core Team 2017). We conducted analyses both at the genotypic and trait levels. We used the genotypic analyses primarily for comparison with previous studies on female × male interactions explaining sperm precedence patterns, which had all been done at this level (see Introduction). Based on previous studies of *D. melanogaster* that have documented genotypic nontransitivity in sperm precedence (Clark et al. 1999, 2000; Zhang et al. 2013), we explicitly predicted a female × male × male interaction in the fertilization set, as well as in reproductive patterns and events leading to this final outcome. The only exceptions were the number of first‐male sperm still residing in the FRT at remating and the progeny produced up to that point, for which we had no a priori expectations of second‐male effects beyond the timing of remating and therefore omitted all second‐male effects in the model. For each focal variable, we conducted either a linear mixed‐effects model (LMM) with the temporal blocking as a four‐level random effect or, for the proportional data of S_2_, a generalized LMM (GLMM) with a binomial error distribution, a logit link, and an additional observation‐level random effect to account for overdispersion. Each model included the female and first‐male and second‐male genotypes with all two‐ and three‐way interactions as fixed effects, except where only females and first males and their interaction were predicted a priori and so any second‐male contributions were excluded. We used conditional *F* tests (LMM) or Wald *χ* ^2^ tests (GLMM) to test for significant main and interactive genotypic effects.

In a second set of analyses (henceforth “traits analyses”), we examined the interrelationships between the male and female reproductive traits themselves. Due to their complexity and a lack of specific information on how precisely traits should interact to explain focal traits, these analyses were necessarily somewhat exploratory. Thus, instead of null‐hypothesis significance testing based on a priori predictions, we used an information‐theoretic approach to account for model uncertainty and identify the most plausible model(s) (Burnham and Anderson 2002; Grueber et al. 2011; Richards et al. 2011; Symonds and Moussalli 2011). These analyses were again based on (G)LMMs, accounting for genetic nonindependence by including each represented genotype and the female × male × male genotypic combination as random effects (and an observation‐level random effect in GLMMs). For each analysis, we generated a model set with all combinations of predictors and interactions (up to a maximum of three‐way interactions for interpretability) from a global model using the *dredge* function implemented in the *MuMIn* package (Bartón 2017). We ranked these models by their Akaike information criterion with sample size adjustment (AIC_c_; Burnham and Anderson 2002; Grueber et al. 2011; Symonds and Moussalli 2011) and limited our confidence model set to candidates within ΔAIC_c_ ≤ 6 of the best model (Bolker et al. 2009; Richards et al. 2011; Symonds and Moussalli 2011), which largely corresponded to cumulative Akaike weights ≥ 0.95. To reduce the retention of overly complex models, we excluded, using the *nested* function in the *MuMIn* package, those models that simply represented more complex versions (e.g., one additional parameter) of any model with a lower AIC_c_ value (Burnham and Anderson 2002; Richards et al. 2011).

Although the primary goal was to determine which explanatory variables and interactions were represented in the confidence model set and thus likely to contribute to the variation in the focal trait, we additionally calculated the natural (conditional) averages and 95% confidence intervals of their coefficients as well as their relative variable importance across the confidence model set (Burnham and Anderson 2002; Grueber et al. 2011). We standardized all predictors (mean = 0; SD = 0.5) to infer standardized effect sizes (Gelman 2008; Grueber et al. 2011).

Finally, we used piecewise structural equation modeling (or confirmatory path analyses; Shipley 2009) in the R package *piecewiseSEM* (Lefcheck 2016) to visualize how the numerous male and female traits directly or indirectly influence the fertilization set (S_2_ in SR) as a proxy of fitness outcomes (Manier et al. 2010, 2013c). This approach decomposes a network of relationships into the simple or multiple linear regressions for each response and allows for combinations of different model structures such as LMM and GLMM. Each regression is assessed separately before being combined to evaluate the entire structural equation model (Lefcheck 2016).

## Results

### GENOTYPIC ANALYSES

We examined how the different genotypes (six females, six first males, and three second‐males), and particularly any two‐ or three‐way interactions between them, contributed to variation in reproductive parameters (e.g., sperm transferred or stored, timing of female sperm ejection), using (G)LMMs with the four temporally separated blocks as a random factor. We omitted in all analyses females that did not complete the experiment (death or loss), had no recorded remating by day 5, or post hoc showed no evidence of successful sperm transfer during the first mating (e.g., no progeny produced between matings and no first‐male sperm found at remating). These exclusions reduced our sample size from 864 to 744 successful mating trios, but additional missing data (e.g., no ejected mass found) resulted in varying sample sizes between analyses.

**Figure 1:**
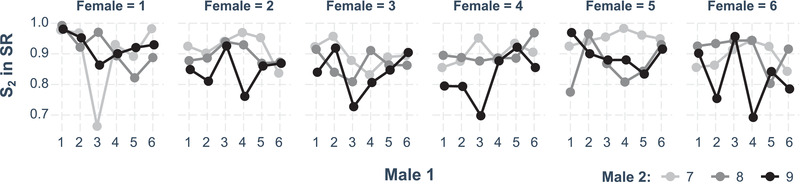
Interactions between female, first‐male and second‐male genotypes explaining variation in the fertilization set. Each dot represents the cross‐specific mean.

We first tested for the contribution of three‐way genotypic interactions to total S_2_ (i.e., the proportional representation of the second‐male's sperm among all sperm retained by the female in both spermathecae and the SR after sperm ejection) and to the fertilization set (i.e., proportional representation in the SR only) (Manier et al. 2010; Lüpold et al. 2012, 2013). In a GLMM with a binomial error distribution and an additional observation‐level random factor to remove overdispersion, variation in total S_2_ was explained by a female × first‐male interaction (*N* = 577; *χ* ^2^_25_ = 39.72, *P* = 0.031) and a weak trend for a three‐way interaction (*χ* ^2^_25_ = 63.50, *P* = 0.095; Table S1). The fertilization set was influenced by a three‐way interaction (*N* = 589; *χ* ^2^_50_ = 69.63, *P* = 0.035), a male × male interaction (*χ* ^2^_10_ = 21.65, *P* = 0.017), and a first‐male main effect (*χ* ^2^_5_ = 16.17, *P* = 0.006; Table S2; Fig. 1).

Next, in LMMs, we focused on two‐ and three‐way interactions between members of the mating trio on specific reproductive traits predicted to contribute to S_2_. We found no interactive effects on traits leading up to the females’ remating (all *P* > 0.16; Tables S3–S5). Rather, the female remating interval, ranging between 2 and 5 days, was explained solely by the female genotype (*F*_5,633.35_ = 6.20, *P* < 0.0001; Table S3). Further, controlling for the remating interval and omitting the second‐male genotypes due to no a priori expectation of their contribution, the number of progeny produced between the first and second mating was influenced primarily by the female genotype (*F*_5,687.32_ = 20.54, *P* < 0.0001), with a weak contribution of the first‐male genotype (*F*_5,687.20_ = 2.11, *P* = 0.063; Table S4). Similarly, accounting for the number of progeny produced as a proxy of sperm use, the number of first‐male sperm still residing in the FRT at remating was also explained by both the female (*F*_5,509.31_ = 10.60, *P* < 0.0001) and first‐male main effects (*F*_5,509.32_ = 2.31, *P* = 0.043; Table S5).

All later reproductive stages exhibited at least some interactive effects, albeit no statistically significant three‐way interaction. For example, the duration of the second copulation was explained primarily by the second‐male's genotype (*F*_5,630.50_ = 55.05, *P* < 0.0001), but also by a female × first‐male interaction (*F*_25,630.61_ = 1.70, *P* = 0.019; Table S6). The number of sperm transferred during these copulations was explained by a trend for a three‐way interaction between all individuals of a mating trio (*F*_50,446.07_ = 1.35, *P* = 0.062) in addition to a strong second‐male main effect (*F*_5,446.04_ = 8.27, *P* = 0.0003; Table S7). Finally, the time to female sperm ejection after remating was determined by the female (*F*_5,537.40_ = 20.99, *P* < 0.0001) and second‐male genotypes (*F*_2,537.41_ = 3.20, *P* = 0.041) and their interaction (*F*_5,537.49_ = 2.20, *P* = 0.017; Table S8).

### TRAITS ANALYSES

In the traits analyses, focusing on the interrelationships between the male and female reproductive traits themselves, we used multimodel inference based on LMMs or GLMMs that included each represented genotype, the female × male × male genotypic combination, and the temporal blocks as random effects. After selecting the confidence model set, we averaged the coefficients using natural model averaging (Burnham and Anderson 2002; Grueber et al. 2011; for details, see Material and Methods).

Our first traits analysis focused on the number of sperm transferred by the second males, which we predicted to depend on female size, copulation duration, the number of first‐male sperm residing within the FRT, and their interactions (Lüpold et al. 2011, 2012). The parsimonious confidence set consisted of two models on second‐male sperm transfer (*N* = 557, ΔAIC_c_ ≤ 0.64), represented by strong positive effects of copulation duration (*β* = 0.26; 95% confidence interval [0.10–0.42]) and the number of resident sperm (*β* = 0.48 [0.32–0.63]), and a weak trend for an interaction between them (*β* = 0.25 [–0.05 to 0.55]; Table S9). These results suggest that males transfer more sperm when there are numerous resident sperm in the FRT, by prolonging copulation. Next, we tested the prediction that the time to female sperm ejection should be influenced by the joint effects of SR length and the differences (second – first male) in sperm length and number between males (*N* = 529). Here, only the difference in sperm length had an effect (*β* = 0.20 [0.05–0.36]; Table S10). Further examination using only the absolute sperm lengths of both males rather than the difference between them revealed that this effect was driven primarily by the second male's sperm length (LMM, *N* = 529; first male: *β* = –0.01 [–0.09 to 0.10]; second‐male: *β* = 0.10 [0.03–0.17]). This result suggests that longer second‐male sperm might prolong the time to female sperm ejection and thus the sperm displacement phase, which was previously shown to increasingly bias sperm storage toward the second male (Lüpold et al. 2013).

**Figure 2:**
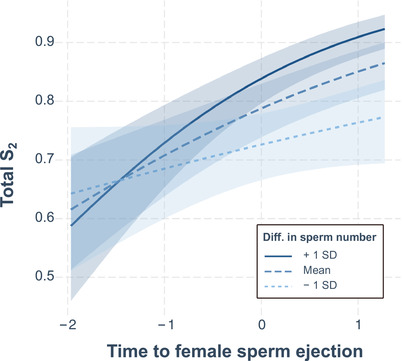
Conditional effects plot of the two‐way interaction between the time from remating to female sperm ejection and the difference (second male – first male) in sperm number explaining variance in total S_2_. The plot indicates that the effects of relative sperm numbers and ejection time reinforce one another, with the greatest change in S_2_ when the second male transfers a disproportionate amount of sperm and the female waits relatively long to eject excess or displaced sperm. The shaded areas around lines depict the 95% confidence intervals.

We further predicted that the relative numbers of first‐ and second‐male sperm stored by females after sperm ejection (i.e., total S_2_) should be explained by the relative sperm lengths and the numbers of first‐male resident and second‐male transferred sperm at the end of copulation (Lüpold et al. 2012), coupled with the timing of female sperm ejection (Lüpold et al. 2013). Using candidate models derived from a GLMM (*N* = 505 observations across all 108 genotypic combinations) including all interactions between ejection time and the between‐male differences in sperm length and numbers, respectively, and with a binomial error distribution and logit link as well as an observation‐level random effect to account for overdispersion, total S_2_ increased with both ejection time (*β* = 0.43 [0.27–0.60]) and the difference in sperm numbers (*β* = 0.69 [0.51–0.86]), and was further influenced by an interaction between these two predictors (*β* = 0.48 [0.16–0.79]; ΔAIC_c_ = 6.99; Table S11 and Fig. 2). This interaction suggests that by delaying or precipitating ejection, females can amplify or dampen, respectively, the competitive advantage of the second male's larger ejaculate.

**Figure 3:**
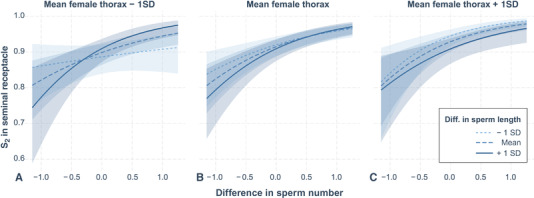
Conditional effects plot of the three‐way interaction between the female thorax length and the relative differences in sperm length and sperm number, respectively, explaining variance in the fertilization set (S_2_ within the female SR). Differences are shown from the second male's perspective (i.e., second male – first male). The plot depicts how any increase in the number of sperm transferred by the second male relative to the first‐male sperm residing in storage is met with a greater change in S_2_ for relatively long sperm in small females (A) but for short sperm in large females (C), with a transitional stage for intermediate female size (B).

**Figure 4:**
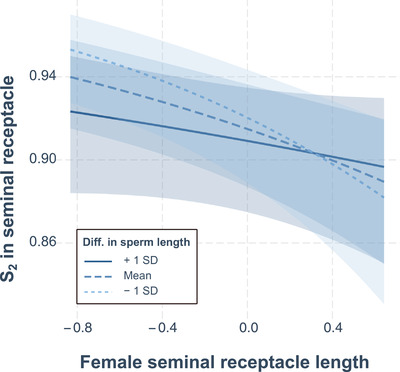
Conditional effects plot of the two‐way interaction between SR length and the difference (second male – first male) in sperm length explaining variance in the fertilization set (S_2_ within the female SR). The plot indicates that the second male's sperm representation in the SR declines with any increase in SR length if his sperm are shorter than those of his rival, but less so when he has relatively longer sperm. The shaded areas around lines depict the 95% confidence intervals.

As mentioned previously, it is important to discriminate between total S_2_ throughout the FRT and the fertilization set in the SR. Here, we repeated the above analysis for S_2_ in the SR, but additionally included SR length and female thorax length as predictors, to examine more complex links between sex‐specific traits explaining relative sperm numbers in the fertilization set. To limit model complexity, we restricted interactions to two‐ and three‐way interactions and further limited all models to a maximum of 10 parameters (including interactions). Although the full model set contained 1294 different models, this was reduced to only 22 models (Table S12) by removing models that were simply more complex versions of any model with a lower AIC_c_ value (Burnham and Anderson 2002; Richards et al. 2011). The resulting confidence model set (ΔAIC_c_ ≤ 6; Bolker et al. 2009; Symonds and Moussalli 2011) consisted of seven models, with female thorax length (*β* = 0.35 [0.11–0.59]), SR length (*β* = –0.56 [–0.79 to –0.32]), the time to sperm ejection (*β* = 0.87 [0.63–1.11]), and difference in the number of sperm between males (*β* = 0.76 [0.51–1.00]) being the most important predictors. The difference in sperm length appeared unimportant as a main effect but contributed to all three interactions whose 95% confidence interval excluded zero after model averaging (Tables 1 and S12). For example, together with female thorax length and the difference in sperm number, it formed a three‐way interaction, meaning that in a small female, any increasing bias in sperm numbers toward the second male will have a strong effect on S_2_ if second male has relatively long sperm, but a weaker effect if he has short sperm (Fig. 3). In large females, however, the effect of relative sperm length on S_2_ tends to reverse. Further, the interaction between the difference in sperm length and SR length (Tables 1 and S12) means that if second males have shorter sperm than their rivals, any increase in SR length reduces S_2_, whereas SR length has a much weaker effect on the fertilization set if second males have relatively longer sperm (Fig. 4). This result corroborates Miller and Pitnick's (2002) findings using populations of *D. melanogaster* with experimentally evolved, exaggerated sperm and SR lengths as well as subsequent studies that also predicted significant effects of interactions between sperm lengths of both males and female SR length on the fertilization set (Pattarini et al. 2006; Lüpold et al. 2013, 2016).

**Table 1:** Model‐averaged coefficients of the analysis on the fertilization set (i.e., S_2_ within the female SR) following sperm ejection, including the standardized effects of the difference (second – first male) in sperm length (ΔSL) and in sperm number (ΔSN), the time to female sperm ejection (EJT), female SR length (SRL), and female thorax length (FTL). *N* = 508, including all 108 genotypic combinations. See Table S12 for full details

Parameter	Estimate	SE	95% CI
Intercept	2.35	0.17	(2.02, 2.68)
ΔSN	0.76	0.12	(0.51, 1.00)
EJT	0.87	0.12	(0.63, 1.11)
SRL	−0.56	0.12	(−0.79, −0.32)
FTL	0.35	0.12	(0.11, 0.59)
ΔSL × FTL	−0.55	0.24	(−1.02, −0.09)
ΔSL	–0.10	0.17	(–0.43, 0.22)
ΔSN × EJT	0.40	0.25	(–0.09, 0.88)
ΔSL × SRL	0.48	0.24	(0.02, 0.94)
ΔSL × ΔSN × FTL	−1.12	0.47	(−2.03, −0.21)
ΔSN × FTL	0.21	0.24	(–0.26, 0.69)
ΔSL × ΔSN	0.18	0.25	(–0.31, 0.66)

### STRUCTURAL EQUATION MODEL

**Figure 5:**
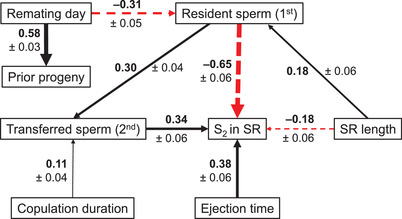
Visual representation of the confirmatory path analysis examining direct and indirect effects on the fertilization set (i.e., S_2_ within the female seminal receptacle). The width of each arrow is proportional to its corresponding coefficient (in bold, ± standard error). Black, solid arrows depict positive effects, whereas red, dashed arrows represent negative effects. All effects shown were statistically significant (*P* ≤ 0.04); all others (*P* ≥ 0.09) are omitted for better visibility (see Fig. S1 for all relationships examined). This also includes any paths involving the lengths of first‐ and second‐male sperm and the female thorax, which had significant effects within some of the individual models but not after combining models.

Finally, we used a piecewise structural equation modeling approach to better visualize both direct and indirect effects of these numerous male and female traits on one another, and particularly on the fertilization set. This analysis revealed a complex network of interrelationships (Fig. 5; see Fig. S1 for visualization of the individual models). Not surprisingly, relative sperm numbers in the FRT immediately after copulation were a good predictor of relative sperm numbers in the SR after ejection, confirming previous results (Lüpold et al. 2012, 2013). Interestingly, longer SRs indirectly decreased S_2_ by storing more resident sperm at the time of remating, contrasting with previous studies suggesting that longer SRs are associated with increased S_2_, particularly when the second male has longer sperm (Miller and Pitnick 2002). However, as shown in the simpler models above, it seems likely that SR length interacts with other reproductive traits, and as a result, can affect S_2_ both positively and negatively depending on the context (note that for simplicity, interactions between traits are omitted in our structural equation models).

## Discussion

There is a growing paradigm in sexual selection theory that emphasizes interactions between the sexes (e.g., Clark 2002; Arnqvist and Rowe 2005; Ravi Ram and Wolfner 2007; Howard et al. 2009; Arnqvist 2014). Advancing such theory is currently limited by our understanding of genotypic interactions between the sexes and of concomitant functional interactions among those sex‐specific traits that impact competitive reproductive outcomes. Here, we identified PSS mechanisms that involve female × male interactions and resolved sex‐specific traits that underlie those mechanisms. We note that these findings are conservative, as with only six respective first‐male and female genotypes and three second‐male genotypes, we worked with limited genetic variation. For logistical reasons, our study also excluded a number of sex‐specific traits that are expected to contribute to PSS, including genitalic traits (e.g., House and Simmons 2003; Kamimura 2005; Wojcieszek and Simmons 2011; Simmons and Fitzpatrick 2019), FRT secretions (Gasparini and Pilastro 2011; Alonzo et al. 2016; Rosengrave et al. 2016; Lehnert et al. 2017), as well as male seminal‐fluid proteins (Clark et al. 1995; Chapman et al. 2000; Nakadera et al. 2014) and their female receptors (Sirot and Wolfner 2015; McDonough et al. 2016). Hence, although this study represents important progress toward understanding the targets and selective dynamics of PSS, there is much more work to be done. It is also important to note that experimental investigation of interactions such as these is analytically complex, every currently available approach having its own limitations and challenges. Although our different statistical approaches (i.e., null‐hypothesis significance testing, information theoretic using AIC‐based multi‐model inference, and path analyses) resulted in similar patterns, there were quantitative differences in the degree to which female × male interactions explained variation in reproductive outcomes. Because there was some phenotypic variation within the heterozygous F_1_ genotypes for all traits examined, and we had only few genotypes, we expect the “traits analyses” to be more sensitive to detecting interactions than the genotypic analyses.

Our investigation corroborated previous work in *D. melanogaster* showing that reproductive outcomes are mediated by male‐specific (Clark et al. 1995; Fiumera et al. 2005; Lüpold et al. 2012; Civetta and Ranz 2019), female‐specific (Clark and Begun 1998; Lüpold et al. 2013; Ala‐Honkola and Manier 2016; Chen et al. 2019), and interactive effects between the sexes and competing males (Clark et al. 1999; Mack et al. 2002; Miller and Pitnick 2002; Bjork et al. 2007; Reinhart et al. 2015), patterns that have also been studied extensively in a diversity of taxa (reviewed in Arnqvist and Rowe 2005; Snook 2005; Oh and Badyaev 2006; Howard et al. 2009; Pitnick et al. 2009b; Pizzari and Parker 2009; Arnqvist 2014; Firman et al. 2017). In support of these interactive effects on relative paternity shares (P_2_) among genotypes, we found that a three‐way interaction between the different genotypes of a mating trio significantly contributed to variation in the fertilization set, which is a consequence of all other traits examined (e.g., the number of sperm transferred by first males, retained and used by females, the number of sperm transferred by second males, and the outcome of sperm displacement activity following remating) and arguably the strongest correlate of variation in male and female fitness (Lüpold et al. 2012, 2013; Manier et al. 2013a,c). This interaction likely explains the previously reported nontransitivity in P_2_ (Clark et al. 1999, 2000; Zhang et al. 2013; Reinhart et al. 2015). Importantly, our analyses allowed us to decompose the underlying processes leading to this outcome, which we here discuss in turn.

The remating interval of females, which is arguably one of the strongest determinants of the intensity of PSS (Boorman and Parker 1976; Simmons 2001; Taylor et al. 2014), was mediated by females alone in our analysis, which might be due to unusually little variation, with 55% of all remating events (410 of 744) occurring 2 days after the first mating, regardless of genotypic combination, and another 26% (197 of 744) on the third day. However, both the number of progeny produced by the female before remating and the number of resident, first‐male sperm remaining in storage at the time of remating were influenced by the female genotype and at least a weak first‐male effect. This female effect is unsurprising and could be the result of variation in female fecundity, sperm retention or sensitivity to seminal‐fluid proteins after the initial mating, or fertilization efficiency (Pitnick et al. 2001; Pischedda et al. 2012; Lüpold et al. 2013; Delbare et al. 2017). The male effect might be attributable to variation in first‐male ejaculate size or seminal fluid composition (Avila et al. 2011; Lüpold et al. 2012). The lack of an interactive effect on female fecundity is consistent with Pischedda et al.’s (2012) study, but contrasts with Delbare et al.’s (2017) study, both of which crossed inbred lines of the same five geographically (and genetically) distant populations of *D. melanogaster* in a 5 × 5 factorial design. Hence, insufficient genetic variation is unlikely to be the only explanation for the absence of contributing interactions in our study.

The duration of the second copulation was determined predominantly by the genotype of the copulating male, with a significant female × first‐male interaction. Male control of copulation duration has been reported in multiple arthropod species (e.g., Yasui 1994; Wilder and Rypstra 2007; Holwell 2008), including *Drosophila* (e.g., MacBean and Parsons 1966, 1967), and is often related to ejaculate transfer and ultimately competitive fertilization success (Parker 1970b; Dickinson 1986; Wolf et al. 1989; García‐González and Gomendio 2004; Wang et al. 2008; reviewed in Weggelaar et al. 2019). The female × first‐male interaction might be related to the number of first‐male sperm residing within the FRT, in that females with a long SR tended to store more first‐male sperm at the time of remating. These patterns combined might then explain the trend for a three‐way interaction in second‐male sperm transfer, with the number of sperm transferred by second males increasing with both the duration of their copulation and the number of first‐male sperm still residing within the female, including a weak interaction between them (Table S9). That male *D. melanogaster* adjust their ejaculate size to the presence or absence of a competitor's sperm has previously been documented (Lüpold et al. 2011) and is a taxonomically widespread phenomenon (meta‐analyzed in Kelly and Jennions 2011). Our results suggest that sperm allocation might be even more sophisticated than previously realized by responding to the quantity of rival sperm in the FRT, even though the mechanism(s) underlying such nuanced adjustments remain(s) elusive.

After copulation, the timing of female ejection was explained by both the female and second‐male genotype, including their interaction. Females that eject sperm later allow more time for second‐male sperm to enter storage and further displace first‐male sperm. Because longer sperm are better at displacing, and resisting displacement by, shorter sperm (Miller and Pitnick 2002; Pattarini et al. 2006; Lüpold et al. 2013; Manier et al. 2013b), longer ejection times benefit males with longer sperm and perhaps also the female through indirect fitness benefits (Lüpold et al. 2016). Indeed, our traits analysis revealed that the difference in sperm length between competing males influenced the timing of female sperm ejection (Table S10), while further engaging in interactions with female size, SR length, and the difference in sperm number to explain a significant proportion of the variance in the fertilization set (Tables S11 and S12). Although the mechanism(s) underlying the delay in female sperm ejection after mating with a long‐sperm male remain(s) unknown, this pattern, combined with a genetic correlation between female sperm ejection and SR length (Lüpold et al. 2016), provides a possible functional explanation for the heightened precedence of relatively long sperm in a long compared to short SR reported previously (Miller and Pitnick 2002).

Because sperm ejection is a principal means by which females influence paternity in *Drosophila* (Snook and Hosken 2004; Manier et al. 2010, 2013a,b; Lüpold et al. 2013) and many other taxa (reviewed in Dean et al. 2011), these results provide strong support for the expectation that cryptic female choice processes will evolve mechanisms entailing interactions between mating partners (Arnqvist 2014; Firman et al. 2017).

The present study examined only a small proportion of the sex‐specific phenotypes suspected of influencing competitive fertilization success. Nevertheless, all measured male and female reproductive traits contributed to the competitive fertilization set or at least to some reproductive event known to determine it. Further, interactions identified here between competing males and between sexes were shown to explain a significant amount of variation in several key reproductive events, including those generally considered functional components of both sperm competition (e.g., the number of sperm inseminated during remating by a female) and cryptic female choice (e.g., sperm ejection time). Moreover, sperm length, which is recognized as a PSS ornament that interacts functionally and evolutionarily with SR length (Miller and Pitnick 2002; Pattarini et al. 2006; Lüpold et al. 2016), did so in a consistent manner in the present study and was further shown to interact with sperm number and SR length in determining competitive fertilization success (i.e., the fertilization set). Our results thus provide further evidence that sperm competition, oftentimes considered to operate between males alone, may in fact rarely be independent of female effects (Eberhard 1996; Arnqvist 2014; Lüpold et al. 2016; Firman et al. 2017), thereby supporting the idea that sperm competition and cryptic female choice are likely to represent a false dichotomy. Because the identified interactions included sperm and SR length, which have been shown to represent one of the most extreme co‐diversifying systems of male ornamentation and female preference (Lüpold et al. 2016), multivariate systems with complex interactions between the sexes might not be limited in their ability to respond to directional sexual selection. This scenario would be the case particularly if these interactions are also context dependent (e.g., due to condition‐dependent ejaculate composition or female sperm‐use dynamics), such that even antagonistic effects on S_2_ between traits (e.g., see Fig. 5) do not necessarily restrict the opportunity for selection on, and thus the evolution of, sex‐specific traits. This raises the question of whether the evolution of extreme phenotypes under directional selection, possibly reflected by the widespread sex‐specific main effects in our analyses, has been facilitated by the combination of limited nontransitivity between genotypes and a complex interplay between sex‐specific, heritable, and likely condition‐dependent traits, which might have helped maintain considerable genetic variation within populations. Finally, our study illustrates both the benefits and empirical challenges of quantifying the contribution of interactions to the operation of sexual selection.

## AUTHOR CONTRIBUTIONS

SL, MKM, JMB, and SP conceived the research. SL, JBR, MKM, VZ, and SP performed the research. SL analyzed the data. SL and SP drafted the manuscript, which all authors edited and approved.

## CONFLICT OF INTEREST

The authors declare no conflict of interest.

## DATA ARCHIVING

All data are deposited in the Dryad Repository (https://doi.org/10.5061/dryad.x3ffbg7gq).

## Supporting information


**Supplementary Figure S1**: Visual representation of the GLMM models used in the confirmatory path analysis, with arrows connecting all predictors with the response variable examined.
**Supplementary Table S1**: Results of a generalized linear mixed model with binomial error distribution representing the genotypic effects on total S_2_ after female sperm ejection (*N* = 577 across all 108 genotypic mating combinations).
**Supplementary Table S2**: Results of a generalized linear mixed model with binomial error distribution representing the genotypic effects on S_2_ within the seminal receptacle (i.e., the “fertilization set”) after female sperm ejection (*N* = 589 across all 108 genotypic mating combinations).
**Supplementary Table S3**: Results of a linear mixed‐effects model (with the temporal block as a four‐level random factor) analyzing the genotypic effects and interactions on the female remating interval.
**Supplementary Table S4**: Results of a linear mixed‐effects model (with the temporal block as a four‐level random factor) analyzing the genotypic effects and interactions on the number of offspring produced between copulations, controlling for the female remating interval.
**Supplementary Table S5**: Results of a linear mixed‐effects model (with the temporal block as a four‐level random factor) analyzing the genotypic effects and interactions on the number of 1st‐male sperm residing in the female reproductive tract at remating, controlling for the number of progeny produced prior to remating.
**Supplementary Table S6**: Results of a linear mixed‐effects model (with the temporal block as a four‐level random factor) analyzing the genotypic effects and interactions on the duration of the second copulation.
**Supplementary Table S7**: Results of a linear mixed‐effects model (with the temporal block as a four‐level random factor) analyzing the genotypic effects and interactions on the number of sperm transferred by the second male.
**Supplementary Table S8**: Results of a linear mixed‐effects model (with the temporal block as a four‐level random factor) analyzing the genotypic effects and interactions on the time to female sperm ejection.
**Supplementary Table S9**: Results of the information‐theoretic analyses examining the effects of copulation duration (C), the number of 1st‐male sperm residing in the FRT at remating (R) and female thorax length (T) on the number of sperm transferred by the second male (N = 557).
**Supplementary Table S10**: Results of the information‐theoretic analyses examining the effects of the difference in sperm length (L), the difference in sperm number (N) and seminal receptacle length (S) on the time to female sperm ejection (N = 527).
**Supplementary Table S11**: Results of the information‐theoretic analyses examining the effects of the difference in sperm length (L), the difference in sperm number (N) and the time to female sperm ejection (E) on the relative numbers of sperm stored between males (i.e., total S_2_, *N* = 505).
**Supplementary Table S12**: Results of the information‐theoretic analyses examining the effects of the difference in sperm length (L), the difference in sperm number (N), the time to female sperm ejection (E), female SR length (S) and female thorax length (T) on S_2_ within the SR (*N* = 508). Each model was a GLMM with the temporal block as a four‐level random factor. An observation‐level random effect was included to account for overdispersion.
