# Supplementary material for: How female × male and male × male interactions influence competitive fertilization in Drosophila melanogaster
Source: Evol Lett. 2020 Sep 4;4(5):416–29. doi: 10.1002/evl3.193 (PMC7523561; doi:10.1002/evl3.193)
Supplement: Supplementary file 1 — Supplementary Figure S1: Visual representation of the GLMM models used in the confirmatory path analysis, with arrows connecting all predictors with the response variable examined. Supplementary Table S1: Results of a generalized linear mixed model with binomial error distribution representing the genotypic effects on total S2 after female sperm ejection (N = 577 across all 108 genotypic mating combinations). Supplementary Table S2: Results of a generalized linear mixed model with binomial error distribution representing the genotypic effects on S2 within the seminal receptacle (i.e., the “fertilization set”) after female sperm ejection (N = 589 across all 108 genotypic mating combinations). Supplementary Table S3: Results of a linear mixed‐effects model (with the temporal block as a four‐level random factor) analyzing the genotypic effects and interactions on the female remating interval. Supplementary Table S4: Results of a linear mixed‐effects model (with the temporal block as a four‐level random factor) analyzing the genotypic effects and interactions on the number of offspring produced between copulations, controlling for the female remating interval. Supplementary Table S5: Results of a linear mixed‐effects model (with the temporal block as a four‐level random factor) analyzing the genotypic effects and interactions on the number of 1st‐male sperm residing in the female reproductive tract at remating, controlling for the number of progeny produced prior to remating. Supplementary Table S6: Results of a linear mixed‐effects model (with the temporal block as a four‐level random factor) analyzing the genotypic effects and interactions on the duration of the second copulation. Supplementary Table S7: Results of a linear mixed‐effects model (with the temporal block as a four‐level random factor) analyzing the genotypic effects and interactions on the number of sperm transferred by the second male. Supplementary Table S8: Results of a linear mixed‐effects model (wit [file EVL3-4-416-s001.pdf]

Supplementary Online Material to:

How female × male and male × male interactions influence competitive fertilization in *Drosophila melanogaster*

Stefan Lüpold[1,2]*, Jonathan Bradley Reil[3,4], Mollie K. Manier[2,5], Valérian Zeender[1], John M. Belote[2] & Scott Pitnick[2]

[1]Department of Evolutionary Biology and Environmental Studies, University of Zurich, 8057 Zurich, Switzerland

[2]Department of Biology, Syracuse University, Syracuse, NY 13244-1270, USA

[3]Department of Entomology, Cornell University, Ithaca, NY 14853, USA

[4]Department of Plant and Environmental Protection Sciences, University of Hawaii at Manoa, Honolulu, Hawaii

[5]Department of Biological Sciences, George Washington University, Washington, DC 20052, USA

*Corresponding author: stefan.luepold@ieu.uzh.ch

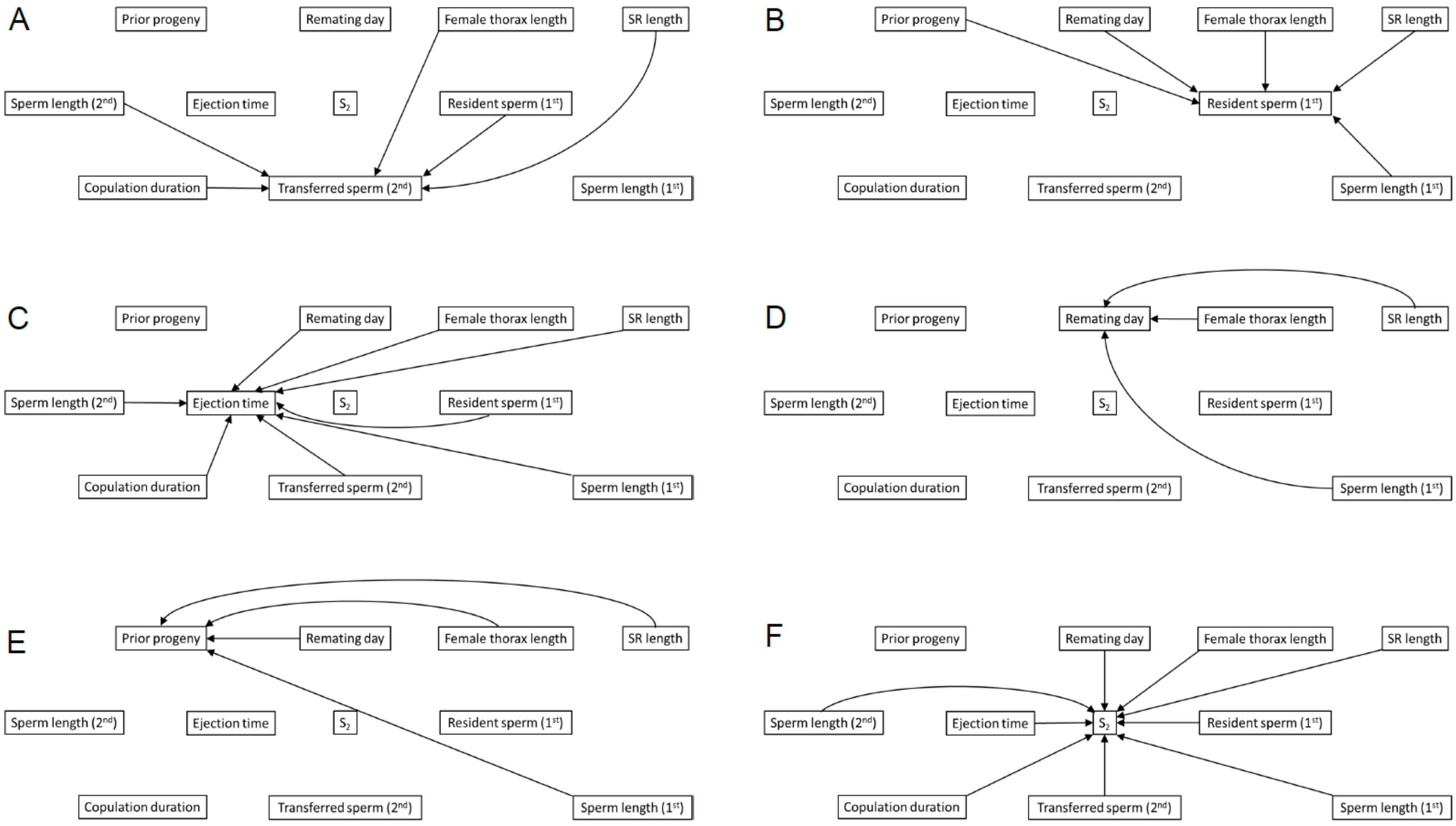

Supplementary Figure S1: Visual representation of the GLMM models used in the confirmatory path analysis, with arrows connecting all predictors with the response variable examined. These individual models were then combined to evaluate the entire structural equation model (see Fig. 1 in the main text).

Supplementary Table S1: Results of a generalized linear mixed model with binomial error distribution representing the genotypic effects on total S_2 after female sperm ejection ($N = 577$ across all 108 genotypic mating combinations). The model includes as a four-level random factor controlling for the temporal experimental blocks and an observation-level random effect (OLRE) to account of overdispersion (final dispersion = 1.05).

Random effects:

Groups	Variance	SD
OLRE	0.57	0.76
Block	<0.01	0.01

Fixed effects:

Predictor	χ^2	*df*	*P*
**Female**	**36.80**	**5**	**<0.001**
**Male 1**	**27.30**	**5**	**<0.001**
**Male 2**	**29.97**	**2**	**<0.001**
**Female × male 1**	**39.99**	**25**	**0.029**
Female × male 2	7.23	10	0.704
Male 1 × male 2	10.28	10	0.416
Female × male 1 × male 2	62.39	50	0.112

Conditional $R^2 = 0.98$, marginal $R^2 = 0.28$

Supplementary Table S2: Results of a generalized linear mixed model with binomial error distribution representing the genotypic effects on S_2 within the seminal receptacle (i.e., the "fertilization set") after female sperm ejection ($N = 589$ across all 108 genotypic mating combinations). The model includes as a four-level random factor controlling for the temporal experimental blocks and an observation-level random effect (OLRE) to account of overdispersion (final dispersion = 1.08). The statistical significance of individual terms was determined using Type III Wald χ^2 tests.

Random effects:

Groups	Variance	SD
OLRE	1.33	1.15
Block	0.02	0.16

Fixed effects:

Predictor	χ^2	*df*	*P*
(Intercept)	36.99	1	<0.001
Female	8.01	5	0.156
**Male 1**	**16.17**	**5**	**0.006**
Male 2	2.20	2	0.333
Female × male 1	34.84	25	0.091
Female × male 2	15.73	10	0.108
**Male 1 × male 2**	**21.65**	**10**	**0.017**
**Female × male 1 × male 2**	**69.63**	**50**	**0.035**

Conditional $R^2 = 0.99$, marginal $R^2 = 0.29$

Supplementary Table S3: Results of a linear mixed-effects model (with the temporal block as a four-level random factor) analyzing the genotypic effects and interactions on the female remating interval. The denominator degrees of freedom (Den DF) were approximated using Kenward-Roger's method (total $N = 744$ across all 108 genotypic combinations).

Random effects:

Groups	Variance	SD
Block	0.01	0.12
Residual	0.76	0.87

Fixed effects:

Terms	Sum Sq	Mean Sq	Num DF	Den DF	*F*	*P*
**Female**	**23.47**	**4.69**	**5**	**633.35**	**6.20**	**<0.001**
Male1	5.38	1.08	5	633.24	1.42	0.215
Male2	2.59	1.30	2	633.21	1.71	0.181
Female × Male 1	24.34	0.97	25	633.22	1.29	0.161
Female × Male 2	10.72	1.07	10	633.32	1.42	0.169
Male 1 × Male 2	2.83	0.28	10	633.21	0.37	0.958
Female × Male 1 × Male 2	29.03	0.58	50	633.21	0.77	0.879

Conditional $R^2 = 0.16$, marginal $R^2 = 0.15$

Supplementary Table S4: Results of a linear mixed-effects model (with the temporal block as a four-level random factor) analyzing the genotypic effects and interactions on the number of offspring produced between copulations, controlling for the female remating interval. Note that second males are omitted because they had no effect on the remating interval (Table S3) as the only way they would be able to influence the number of progeny sired by the first male. The denominator degrees of freedom (Den DF) were approximated using the using Kenward-Roger's method (total $N = 726$ across all 108 genotypic combinations).

Random effect:

Groups	Variance	SD
Block	0.00	0.00
Residual	1085.00	32.94

Fixed effects:

Terms	Sum Sq	Mean Sq	Num DF	Den DF	*F*	*P*
**Female**	**111401**	**22280**	**5**	**687.32**	**20.54**	**<0.001**
Male1	11441	2288	5	687.20	2.11	0.063
**Remating interval**	**320684**	**320684**	**1**	**663.54**	**295.57**	**<0.001**
Female × Male 1	27320	1093	25	686.82	1.01	0.454

Conditional $R^2 = 0.39$, marginal $R^2 = 0.39$

Supplementary Table S5: Results of a linear mixed-effects model (with the temporal block as a four-level random factor) analyzing the genotypic effects and interactions on the number of 1st-male sperm residing in the female reproductive tract at remating, controlling for the number of progeny produced prior to remating. Note that 2nd males are omitted because they had no effect on the remating interval (Table S3) as the only way they would be able to influence the number of 1st-male sperm remaining in storage. The denominator degrees of freedom (Den DF) were approximated using Kenward-Roger's method (total N = 549 across all 108 genotypic combinations).

Random effects:

Groups	Variance	SD
Block	909.7	30.16
Residual	22477.1	149.92

Fixed effects:

Terms	Sum Sq	Mean Sq	Num DF	Den DF	*F*	*P*
**Female**	**1191684**	**238337**	**5**	**509.31**	**10.60**	**<0.001**
**Male1**	**259168**	**51834**	**5**	**509.32**	**2.31**	**0.043**
**Prior progeny**	**359727**	**359727**	**1**	**510.16**	**16.00**	**<0.001**
Female × Male 1	620244	24810	25	509.33	1.10	0.333

Conditional R^2 = 0.18, marginal R^2 = 0.15

Supplementary Table S6: Results of a linear mixed-effects model (with the temporal block as a four-level random factor) analyzing the genotypic effects and interactions on the duration of the second copulation. The denominator degrees of freedom (Den DF) were approximated using Kenward-Roger's method (total N = 741 across all 108 genotypic combinations).

Random effects:

Groups	Variance	SD
Block	0.09	0.30
Residual	52.13	7.22

Fixed effects:

Terms	Sum Sq	Mean Sq	Num DF	Den DF	*F*	*P*
Female	209.4	41.9	5	631.01	0.80	0.547
Male1	74.5	14.9	5	630.84	0.29	0.921
**Male2**	**5738.9**	**2869.5**	**2**	**630.50**	**55.05**	**<0.001**
**Female × Male 1**	**2213.6**	**88.5**	**25**	**630.61**	**1.70**	**0.019**
Female × Male 2	461.6	46.2	10	630.98	0.89	0.546
Male 1 × Male 2	444.9	44.5	10	630.62	0.85	0.577
Female × Male 1 × Male 2	2685.5	53.7	50	630.54	1.03	0.419

Conditional R^2 = 0.24, marginal R^2 = 0.24

Supplementary Table S7: Results of a linear mixed-effects model (with the temporal block as a four-level random factor) analyzing the genotypic effects and interactions on the number of sperm transferred by the second male. The denominator degrees of freedom (Den DF) were approximated using Kenward-Roger's method (total $N = 557$ across all 108 genotypic combinations).

Random effects:

Groups	Variance	SD
Block	30207	173.8
Residual	153218	391.4

Fixed effects:

Terms	Sum Sq	Mean Sq	Num DF	Den DF	*F*	*P*
Female	907851	181570	5	446.08	1.19	0.315
Male1	668134	133627	5	446.08	0.87	0.500
**Male2**	**2533620**	**1266810**	**2**	**446.04**	**8.27**	**<0.001**
Female × Male 1	5000559	200022	25	446.09	1.31	0.149
Female × Male 2	986354	98635	10	446.08	0.64	0.776
Male 1 × Male 2	2395496	239550	10	446.09	1.56	0.115
**Female × Male 1 × Male 2**	**10342191**	**206844**	**50**	**446.07**	**1.35**	**0.062**

Conditional $R^2 = 0.32$, marginal $R^2 = 0.19$

Supplementary Table S8: Results of a linear mixed-effects model (with the temporal block as a four-level random factor) analyzing the genotypic effects and interactions on the time to female sperm ejection. The denominator degrees of freedom (Den DF) were approximated using Kenward-Roger's method (total $N = 648$ across all 108 genotypic combinations).

Random effects:

Groups	Variance	SD
Block	0.004	0.061
Residual	0.170	0.412

Fixed effects:

Terms	Sum Sq	Mean Sq	Num DF	Den DF	*F*	*P*
**Female**	**17.81**	**3.56**	**5**	**537.40**	**20.99**	**<0.001**
Male1	0.15	0.03	5	537.51	0.18	0.970
**Male2**	**1.09**	**0.54**	**2**	**537.41**	**3.20**	**0.042**
Female × Male 1	4.49	0.18	25	537.36	1.06	0.386
**Female × Male 2**	**3.73**	**0.37**	**10**	**537.49**	**2.20**	**0.017**
Male 1 × Male 2	1.29	0.13	10	537.47	0.76	0.669
Female × Male 1 × Male 2	5.82	0.12	50	537.30	0.69	0.951

Conditional $R^2 = 0.25$, marginal $R^2 = 0.23$

Supplementary Table S9: Results of the information-theoretic analyses examining the effects of copulation duration (C), the number of 1st-male sperm residing in the FRT at remating (R) and female thorax length (T) on the number of sperm transferred by the second male (N = 557). Each model was a linear mixed-effects model with the temporal block as a four-level random factor. The last two columns list the results when slightly more complex, nested versions of higher-ranked models are excluded, following the recommendation of Burnham and Anderson (2002) and Richards et al. (2011). The most parsimonious confidence model set is highlighted in bold.

Model	*df*	logLik	AIC_c	ΔAIC_c	w_i	cum w_i	ER	ΔAIC_c	w_i
**C + R + C×R**	**10**	**-734.19**	**1488.78**	**0.00**	**0.30**	**0.30**	**—**	**0.00**	**0.30**
**C + R**	**9**	**-735.55**	**1489.42**	**0.64**	**0.23**	**0.53**	**1.30**	**0.64**	**0.23**
C + R + T + C×R	11	-734.01	1490.51	1.72	0.13	0.66	2.31		
C + R + T	10	-735.36	1491.13	2.34	0.09	0.75	3.33		
C + R + T + C×R + R×T	12	-733.71	1492.00	3.21	0.06	0.81	5.00		
C + R + T + C×R + C×T	12	-733.84	1492.26	3.48	0.05	0.86	6.00		
C + R + T + R×T	11	-735.06	1492.60	3.82	0.04	0.90	7.50		
C + R + T + C×T	11	-735.14	1492.77	3.99	0.04	0.94	7.50		
C + R + T + C×R + C×T + R×T	13	-733.55	1493.76	4.98	0.02	0.96	15.00		
C + R + T + C×T + R×T	12	-734.84	1494.26	5.47	0.03	0.99	10.00		
C + R + T + C×R + C×T + R×T + C×R×T	14	-733.29	1495.35	6.57	0.01	1.00	30.00		
R	8	-740.51	1497.29	8.50	0.00	1.00	∞	8.50	0.00
R + T	9	-740.39	1499.11	10.32	0.00	1.00	∞		
R + T + R×T	10	-740.09	1500.58	11.79	0.00	1.00	∞		
C	8	-753.37	1523.01	34.22	0.00	1.00	∞	34.22	0.00
C + T	9	-753.36	1525.05	36.27	0.00	1.00	∞		
C + T + C×T	10	-753.14	1526.67	37.89	0.00	1.00	∞		
(Null)	7	-757.47	1529.14	40.35	0.00	1.00	∞	40.35	0.00
T	8	-757.47	1531.19	42.41	0.00	1.00	∞		

Coefficients of the above analyses (naturally) averaged across the reduced confidence model set, including the conditional standard errors (SE) and 95% confidence limits (CL), the relative variable importance (*RI*) and number of models including each term (*N*).

	Full confidence set					Excluding nested models				
**Parameter**	**Estimate**	**SE**	**95% CL**	***RI***	***N***	**Estimate**	**SE**	**95% CL**	***RI***	***N***
(Intercept)	0.01	0.20	(-0.37, 0.40)		10	0.01	0.20	(-0.37, 0.40)		2
**R**	**0.48**	**0.08**	**(0.32, 0.63)**	**1.00**	**10**	**0.48**	**0.08**	**(0.32, 0.63)**	**1.00**	**2**
**C**	**0.26**	**0.08**	**(0.10, 0.42)**	**1.00**	**10**	**0.26**	**0.08**	**(0.10, 0.42)**	**1.00**	**2**
C×R	0.25	0.15	(-0.05, 0.55)	0.58	5	0.25	0.15	(-0.05, 0.55)	0.58	1
T	0.07	0.11	(-0.15, 0.28)	0.47	8					
R×T	-0.12	0.16	(-0.43, 0.19)	0.15	4					
C×T	-0.10	0.16	(-0.42, 0.22)	0.14	4					

Supplementary Table S10: Results of the information-theoretic analyses examining the effects of the difference in sperm length (L), the difference in sperm number (N) and seminal receptacle length (S) on the time to female sperm ejection (*N* = 527). Each model was a linear mixed-effects model with the temporal block as a four-level random factor. The last two columns list the results when slightly more complex, nested versions of higher-ranked models are excluded, following the recommendation of Burnham and Anderson (2002) and Richards et al. (2011). The most parsimonious confidence model set is highlighted in bold.

Model	*df*	logLik	AIC$_c$	ΔAIC$_c$	w_i	cum w_i	ER	ΔAIC$_c$	w_i
**L**	**8**	**-711.91**	**1440.10**	**0.00**	**0.23**	**0.23**	—	**0.00**	**0.61**
**(Null)**	**7**	**-713.39**	**1441.00**	**0.90**	**0.15**	**0.38**	**1.53**	**0.90**	**0.39**
L + S + L×S	10	-710.62	1441.67	1.57	0.11	0.49	2.09		
L + S	9	-711.83	1442.01	1.91	0.09	0.58	2.56		
L + N	9	-711.88	1442.11	2.01	0.08	0.66	2.88		
S	8	-713.31	1442.90	2.80	0.06	0.72	3.83		
N	8	-713.36	1443.00	2.90	0.05	0.77	4.60		
L + N + L×N	10	-711.46	1443.35	3.25	0.05	0.82	4.60		
L + N + S + L×S	11	-710.57	1443.65	3.55	0.04	0.86	5.75		
L + N + S	10	-711.80	1444.02	3.92	0.03	0.89	7.67		
N + S	9	-713.28	1444.90	4.80	0.02	0.91	11.50		
L + N + S + L×N + L×S	12	-710.21	1445.02	4.92	0.02	0.93	11.50		
L + N + S + L×N	11	-711.38	1445.28	5.18	0.02	0.95	11.50		
L + N + S + L×S + N×S	12	-710.51	1445.63	5.53	0.01	0.96	23.00		
L + N + S + N×S	11	-711.74	1445.98	5.88	0.01	0.97	23.00		
N + S + N×S	10	-713.22	1446.87	6.77	0.01	0.98	23.00		
L + N + S + L×N + L×S + N×S	13	-710.13	1446.96	6.86	0.01	0.99	23.00		
L + N + S + L×N +N×S	12	-711.30	1447.20	7.10	0.01	1.00	23.00		
L + N + S + L×N + L×S + N×S + L×N×S	14	-709.68	1448.18	8.08	0.00	1.00	∞		

Coefficients of the above analyses (naturally) averaged across the reduced confidence model set, including the conditional standard errors (SE) and 95% confidence limits (CL), the relative variable importance (*RI*) and number of models including each term (*N*).

	Full confidence set					Excluding nested models				
**Parameter**	**Estimate**	**SE**	**95% CL**	***RI***	***N***	**Estimate**	**SE**	**95% CL**	***RI***	***N***
(Intercept)	-0.02	0.18	(-0.37, 0.34)	NA	15	-0.02	0.19	(-0.38, 0.35)	0.08	0.94
**L**	**0.20**	**0.08**	**(0.05, 0.36)**	**0.71**	**11**	**0.20**	**0.08**	**(0.05, 0.36)**	**2.56**	**0.01**
S	-0.13	0.32	(-0.75, 0.49)	0.42	10					
N	-0.03	0.09	(-0.19, 0.14)	0.35	4					
L×S	0.25	0.16	(-0.07, 0.56)	0.18	10					
L×N	-0.15	0.17	(-0.48, 0.18)	0.09	3					
N×S	-0.05	0.16	(-0.36, 0.25)	0.03	2					

Supplementary Table S11: Results of the information-theoretic analyses examining the effects of the difference in sperm length (L), the difference in sperm number (N) and the time to female sperm ejection (E) on the relative numbers of sperm stored between males (i.e., total S_2, $N = 505$). Each model was a GLMM with the temporal block as a four-level random factor. An observation-level random effect was included to account for overdispersion. The last two columns list the results when slightly more complex, nested versions of higher-ranked models are excluded, following the recommendation of Burnham and Anderson (2002) and Richards et al. (2011). The most parsimonious confidence model set is highlighted in bold.

Model	*df*	logLik	AIC_c	ΔAIC_c	w_i	cum w_i	ER	ΔAIC_c	w_i
**N + E + N×E**	**10**	**-2952.99**	**5926.42**	**0.00**	**0.38**	0.38	—	**0.00**	**0.95**
L + N + E + L×N + N×E	12	-2951.51	5927.65	1.22	0.21	0.59	1.81		
L + N + E + N×E	11	-2952.60	5927.74	1.32	0.20	0.79	1.90		
L + N + E + L×N + L×E + N×E	13	-2951.48	5929.70	3.28	0.07	0.86	5.43		
L + N + E + L×E + N×E +	12	-2952.58	5929.79	3.37	0.07	0.93	5.43		
L + N + E + L×N + L×E + N×E + L×N×E	14	-2951.22	5931.30	4.88	0.04	0.97	9.50		
L + N + E + L×N	11	-2955.44	5933.41	6.99	0.02	0.99	19.00	6.99	0.03
N + E	9	-2957.68	5933.73	7.30	0.01	1.00	38.00	7.30	0.02
L + N + E	10	-2957.38	5935.20	8.78	0.00	1.00	∞		
L + N + E + L×N + L×E	12	-2955.42	5935.47	9.04	0.00	1.00	∞		
L + N + E + L×E	11	-2957.36	5937.26	10.83	0.00	1.00	∞		
N	8	-2971.05	5958.38	31.96	0.00	1.00	∞	31.96	0.00
L + N + L×N	10	-2968.98	5958.41	31.99	0.00	1.00	∞		
L + N	9	-2970.56	5959.48	33.05	0.00	1.00	∞		
E	8	-2986.81	5989.90	63.48	0.00	1.00	∞	63.48	0.00
L + E	9	-2986.75	5991.87	65.45	0.00	1.00	∞		
L + E + L×E	10	-2986.75	5993.95	67.53	0.00	1.00	∞		
(Null)	7	-2998.32	6010.87	84.45	0.00	1.00	∞	84.45	0.00
L	8	-2998.19	6012.68	86.25	0.00	1.00	∞		

Coefficients of the above analyses (naturally) averaged across the reduced confidence model set, including the conditional standard errors (SE) and 95% confidence limits (CL), the relative variable importance (*RI*) and number of models including each term (*N*).

	Full confidence set					Excluding nested models				
**Parameter**	**Estimate**	**SE**	**95% CL**	***RI***	***N***	**Estimate**	**SE**	**95% CL**	***RI***	***N***
(Intercept)	1.33	0.14	(1.06, 1.60)		6	1.33	0.14	(1.05, 1.61)		6
**N**	**0.69**	**0.09**	**(0.51, 0.86)**	**1.00**	**6**	**0.68**	**0.09**	**(0.51, 0.85)**	**1.00**	**4**
**E**	**0.43**	**0.09**	**(0.27, 0.60)**	**1.00**	**6**	**0.44**	**0.09**	**(0.27, 0.60)**	**1.00**	**4**
**N×E**	**0.48**	**0.16**	**(0.16, 0.79)**	**1.00**	**6**	**0.49**	**0.16**	**(0.18, 0.80)**	**0.95**	**1**
L	0.16	0.16	(-0.15, 0.46)	0.60	5	0.14	0.16	(-0.16, 0.45)	0.03	1
L×N	0.25	0.17	(-0.08, 0.58)	0.33	3	**0.33**	**0.17**	**(0.02, 0.66)**	**0.03**	**1**
L×E	0.03	0.15	(-0.27, 0.34)	0.19	3					
L×N×E	0.23	0.32	(-0.40, 0.85)	0.04	1					

Supplementary Table S12: Results of the information-theoretic analyses examining the effects of the difference in sperm length (L), the difference in sperm number (N), the time to female sperm ejection (E), female SR length (S) and female thorax length (T) on S_2 within the SR ($N = 508$). Each model was a GLMM with the temporal block as a four-level random factor. An observation-level random effect was included to account for overdispersion. Due to many similar models, table only summarizes the data after excluding the slightly more complex, nested versions of higher-ranked models Burnham and Anderson (2002) and Richards et al. (2011). The most parsimonious confidence model set is highlighted in bold.

Model	*df*	logLik	AIC$_c$	ΔAIC$_c$	w_i	cum w_i	ER
**L + N + E + S + T + L×S + L×T + N×E**	**15**	**-2458.91**	**4948.80**	**0.00**	**0.25**	**0.25**	
**L + N + E + S + T + L×S + L×T**	**14**	**-2459.98**	**4948.80**	**0.01**	**0.25**	**0.51**	**1.00**
**L + N + E + S + T + L×N + L×T + N×E + N×T + L×N×T**	**17**	**-2457.23**	**4949.70**	**0.91**	**0.16**	**0.67**	**1.58**
**L + N + E + S + T + L×T + N×E**	**14**	**-2460.93**	**4950.70**	**1.92**	**0.10**	**0.76**	**2.61**
**L + N + E + S + T + L×T**	**13**	**-2462.05**	**4950.80**	**2.04**	**0.09**	**0.85**	**2.78**
**N + E + S + T + N×E**	**12**	**-2463.35**	**4951.30**	**2.53**	**0.07**	**0.92**	**3.56**
**N + E + S + T**	**11**	**-2464.58**	**4951.70**	**2.88**	**0.06**	**0.98**	**4.22**
N + E + S + N×E	11	-2466.78	4956.10	7.29	0.01	0.99	36.14
N + E + S	10	-2467.98	4956.40	7.60	0.01	1.00	42.17
L + N + E + T + L×N + L×T + N×E + N×T + L×N×T	16	-2463.17	4959.40	10.64	0.00	1.00	∞
N + E + N×E	10	-2469.53	4959.50	10.71	0.00	1.00	∞
N + E	9	-2470.77	4959.90	11.09	0.00	1.00	∞
L + E + S + T + L×S + L×T	13	-2477.70	4982.10	33.33	0.00	1.00	∞
E + S + T	10	-2482.03	4984.50	35.70	0.00	1.00	∞
E + S + E×S	10	-2484.91	4990.30	41.47	0.00	1.00	∞
E + S	9	-2486.07	4990.50	41.70	0.00	1.00	∞
E	8	-2489.05	4994.40	45.60	0.00	1.00	∞
L + N + S + T + L×N + L×S + L×T + N×T + L×N×T	16	-2480.76	4994.60	45.84	0.00	1.00	∞
N + S	9	-2488.16	4994.70	45.89	0.00	1.00	∞
N	8	-2492.40	5001.10	52.29	0.00	1.00	∞
S	8	-2503.59	5023.50	74.66	0.00	1.00	∞
(Null)	7	-2508.79	5031.80	83.01	0.00	1.00	∞

Coefficients of the above analyses (naturally) averaged across the reduced confidence model set, including the conditional standard errors (SE) and 95% confidence limits (CL), the relative variable importance (*RI*) and number of models including each term (*N*).

Parameter	Estimate	SE	95% CL	*RI*	*N*
(Intercept)	2.35	0.17	(2.02, 2.68)		7
**N**	**0.76**	**0.12**	**(0.51, 1.00)**	**1.00**	**5**
**E**	**0.87**	**0.12**	**(0.63, 1.11)**	**1.00**	**7**
**S**	**-0.56**	**0.12**	**(-0.79, -0.32)**	**1.00**	**7**
**T**	**0.35**	**0.12**	**(0.11, 0.59)**	**1.00**	**7**
**L×T**	**-0.55**	**0.24**	**(-1.02, -0.09)**	**0.87**	**2**
L	-0.10	0.17	(-0.43, 0.22)	0.87	7
N×E	0.40	0.25	(-0.09, 0.88)	0.59	5
**L×S**	**0.48**	**0.24**	**(0.02, 0.94)**	**0.51**	**4**
**L×N×T**	**-1.12**	**0.47**	**(-2.03, -0.21)**	**0.16**	**1**
N×T	0.21	0.24	(-0.26, 0.69)	0.16	1
L×N	0.18	0.25	(-0.31, 0.66)	0.16	1

References

Burnham, K. P., and D. R. Anderson. 2002. Model Selection and Multi-Model Inference: A Practical Information-Theoretic Approach. 2nd edition. Springer, New York.

Richards, S. A., M. J. Whittingham, and P. A. Stephens. 2011. Model selection and model averaging in behavioural ecology: The utility of the IT-AIC framework. Behav. Ecol. Sociobiol. 65:77–89.
